# Near-infrared magnetic core-shell nanoparticles based on lanthanide metal-organic frameworks as a ratiometric felodipine sensing platform

**DOI:** 10.1038/s42004-023-00893-7

**Published:** 2023-05-18

**Authors:** Yu-Peng Jiang, Xin-Hui Fang, Qian Wang, Jian-Zhong Huo, Yuan-Yuan Liu, Xin-Rui Wang, Bin Ding

**Affiliations:** Tianjin Key Laboratory of Structure and Performance for Functional Molecule, College of Chemistry, Tianjin Normal University, 393 Binshui West Road, Tianjin, 300387 P. R. China

**Keywords:** Coordination chemistry, Metal-organic frameworks, Biosensors, Nanoparticles

## Abstract

Felodipine is an effective drug to treat hypertension, but its abuse can cause bardycardia. It is significant to develop highly sensitive detection platform for felodipine to enable the efficient treatment of hypertension diseases. In this work, to highly efficiently detect felodipine, multi-emission near-infrared (NIR) hierarchical magnetic core-shell lanthanide-MOF nanoparticles, namely Nd-MOF@Yb-MOF@SiO_2_@Fe_3_O_4_ (**NIR-1**), has been synthesized by layer-by-layer (LBL) method. LBL method can adjust the optical properties of **NIR-1** and expose more active sites to improve sensitivity in detection process. **NIR-1** has near-infrared luminescence emission, which can efficiently avoid the interference of autofluorescence in biological tissues. Photo-luminescent (PL) experiments also reveal that **NIR-1** could be used as a near-infrared ratiometric luminescent sensor for felodipine detection with high selectivity and sensitivity, the low of detection limit (LOD) is 6.39 nM in felodipine detection, which is also performed using real biological samples. In addition, **NIR-1** can be used as a ratiometric thermometer could also be applied in the temperature sensing from 293 K to 343 K. Finally, detection mechanisms for felodipine and temperature sensing performance based on near-infrared (NIR) emission were also investigated and discussed in detail.

## Abstract (web summary)

Monitoring of the hypertension drug felodipine is important to avoid unwanted side effects from high concentrations, but its sensitive detection remains challenging. Here, combining the advantages of ratiometric luminescence probes and near-infrared lanthanide luminescence sensing, multi-emission near-infrared magnetic core-shell nanoparticles based on lanthanide metal–organic frameworks are synthesized using a layer-by-layer method and shown to have excellent temperature and felodipine detection abilities.

## Introduction

In the past 30 years, Metal organic frameworks (MOFs) have been widely used in catalysis, gas storage and separation, drug delivery, luminescent sensing and other fields, because these MOFs-based materials possess high porosity, high crystallization, flexible structure motifs^1–10^. In recent years, lanthanide metal organic frameworks (Ln-MOFs) have attracted extensive attentions from researchers. Because of “antenna effect”, Ln-MOFs have excellent optical properties such as long lifetime, high quantum yield and sharp emission peaks^11,12^. Therefore, Ln-MOFs are considered sensing matrices with significant potential applications^13^.

With continuous development of the biological field, traditional MOF-based sensors are also facing urgency challenges such as biological imaging and biosensing. Near-infrared (NIR) luminescent detection has become a rising star because of its ability to cope with these challenges. In 2013, Foucault-Collet et al. successfully designed and synthesized NIR nano Ln-MOF, Yb-PVDC-3. When Yb-PVDC-3 was phagocytosed and internalized, NIR luminescent imaging of living hela cells was realized, and the Yb emission signal can remained stable for 13 h^14^. NIR luminescent sensors have many advantages over visible-light MOFs sensors, for example, large Stokes shift of NIR luminescent can avoid the background fluorescence interference of biological fluids. Additionally it can reduce the photodamage to biological tissue samples. NIR luminescent material has higher vital penetration ability and can better penetrate biological tissues for imaging.

Single emission of Ln-MOFs has poor anti-interference ability, which is easy to be affected by external environment and the ratiometric luminescence probes can deal with this problem well^15,16^. In 2015, Zhang et al. successfully synthesized multi-emission Ln-MOFs, MZMOF-3. It has a self-correcting function and has been successfully used to detect the biomarker for ovarian cancer in serum samples^17^. In 2020, Gomez et al. successfully synthesized NIR multi-emission Ln-MOFs, [LnCl(NDC)(DMF)] (Ln^3+^ = Yb^3+^, Nd^3+^), it has excellent temperature sensing performance in the range of 15-300 K^18^. Ratiometric luminescent probe can resist external environment interference and NIR luminescent probe can avoid autologous fluorescence interference of biological samples. Therefore, considering the above advantages, it is meaningful to construct NIR ratiomeric luminescent probe for biological sample detection and temperature sensing. By detecting optical signals of these NIR, composition analysis of internal environment of organisms can be realized. Non-contact temperature sensing protects biological samples, and nano-scale temperature sensing also has the possibility of application.

Multi-shell MOF@magnetic core nanoparticles synthesized by layer-by-layer (LBL) method have many unique advantages. Firtly, nanoparticles prepared based on spherical magnetic core are also spherical, and it have a larger specific surface area compared with blocky MOFs, which could improve the sensitivity of detection; Secondly, the size of nanoparticles is adjustable, which affects the optical properties of nanoparticles; Thirdly, multi-component MOFs make it possible to customize pore shapes and anisotropic frameworks, enriching the geometry of nanoparticles. Therefore, the Ln-MOFs sensors prepared by this strategy have great application potential^19^. For example^20^, Shi’s and coworkers synthesized magnetic core/multishell Ln-MOF **Fe**_**2**_**O**_**3**_**@SiO**_**2**_**@Eu-MOF@Tb-MOF** (**MagMOF**) nanoballs by layer-by-layer synthesis^20^. **MagMOF** has been successfully used to detect acute myocardial infarction (AMI) biomarkers with high sensitivity, including creatine kinase isoenzyme (CK-MB), troponin I (CTn I), and myoglobin (Mb).

Hypertension is a significant cause of death and disease worldwide, which significantly increases the risk of heart, brain, and kidney disease^21^. According to the World Health Organization (WHO), in the past 30 years, the number of people with high blood pressure worldwide has doubled to 1.4 billion^22^. 0.7 billion patients did not diagnosis timely and suffered from it. The initial treatment of hypertension can effectively control by felodipine, but its abuse can cause excessive peripheral vasodilation with marked hypotension and bardycardia^23^. In order to monitor the concentration of felodipine in patients’ serum, it is the urgent need for developing a novel method to realize anti-hypertensive drugs sensing, which have better therapeutic effect on hypertension. In 2022, Han et al. constructed a ratiometric luminescent sensor by using carbon dots and gold nanoclusters to achieve efficient antihypertensive drug captopril detection^24^. Furthermore, the probe was prepared into a luminescent test strip, and the visual sensing for quantitative detection of captopril was successfully realized with the assistance of a smartphone, the LOD is 101.3 nM. However, to the best of our knowledge, for the detection example for antihypertensive drug felodipine detection still remains a challenge.

**Fig. 1:**
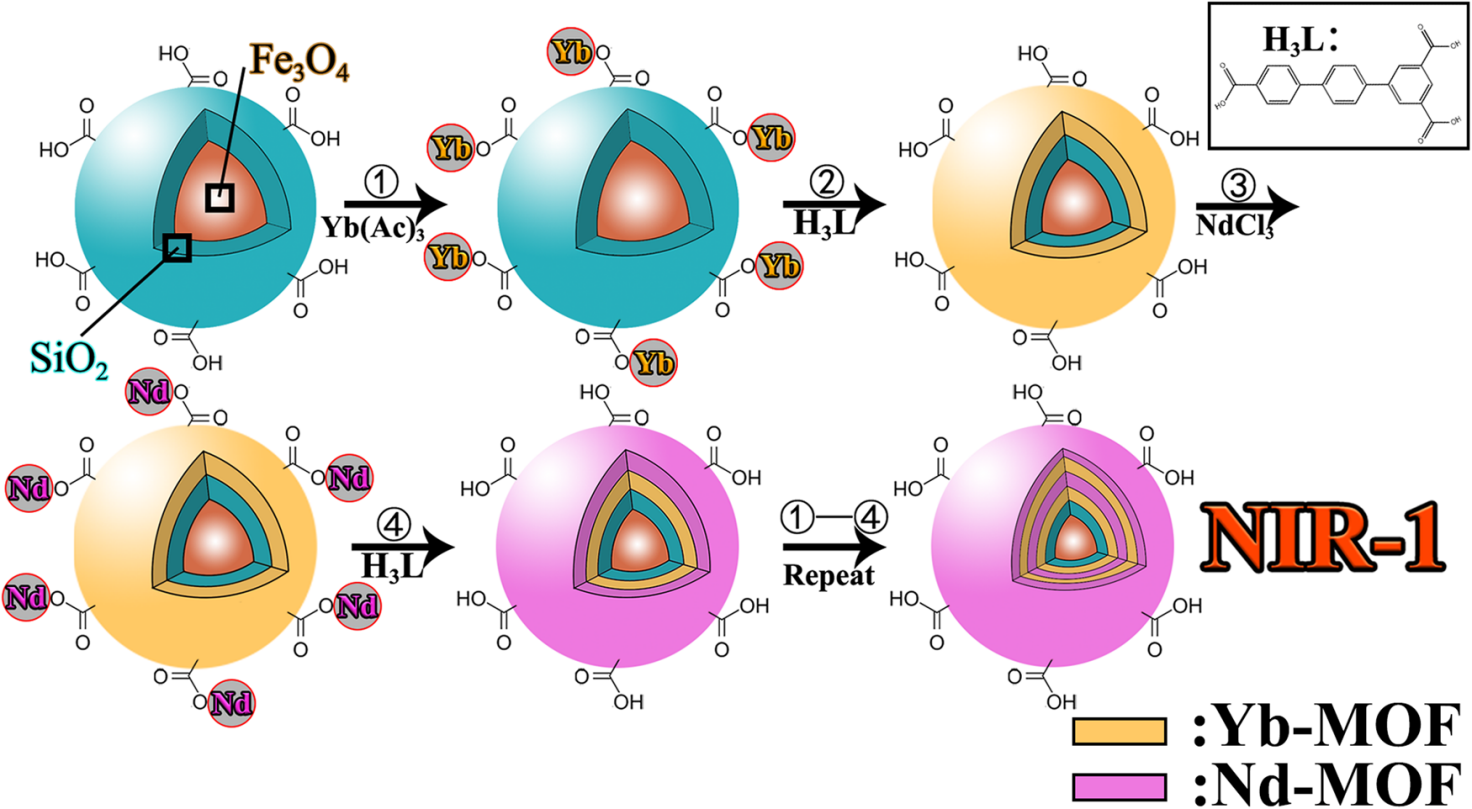
Synthesis method abstract. Preparation of Near-Infrared hierarchical lanthanide-MOF magnetic core-shell nanoparticles Nd-MOF@Yb-MOF@SiO_2_@Fe_3_O_4_ (**NIR-1**).

In this work, considering the advantages of the ratiometric luminescent probe and NIR lanthanide luminescent sensing, we successfully synthesized multi-emission NIR Yb/Nd-MOF by layer-by-layer method (Fig. 1), namely Nd-MOF@Yb-MOF@SiO_2_@Fe_3_O_4_ (**NIR-1**). We apply Fe_3_O_4_@SiO_2_ as template to form nanoball **NIR-1** as the surface of Fe_3_O_4_@SiO_2_ attached with carboxylate group can coordinated with Ln^3+^ ions further to construct MOF layer. Compared with the previous work (**MagMOF**)^20^, **NIR-1** has two innovations. Firstly, the NIR emission probes can be better applied to the biological sample detection. Secondly, a series of **NIR-1** nanoparticles with different properties were synthesized and tested by adjusting the layer of **NIR-1**. Photoluminescence (PL) experiment showed that **NIR-1** could be used as the first near-infrared ratiometric luminescent probe for felodipine with high selectivity and sensitivity, the detection sensitivity parameter is calculated to be 1.4 × 10^7^ [M]^-1^ and LOD is 6.39 nM. Additionally, **NIR-1** also can be applied as a ratiometric luminescent thermometer for temperature sensing. Further, the blind box experiment proved that **NIR-1** could judge the content of mixed drugs (felodipine, bisoprolol and carvedilol) in the solutions through the analysis of luminescence signal response. Finally, the detection mechanisms for felodipine and temperature sensing performance were investigated and discussed in detail.

## Results and discussion

### PXRD patterns, FT-IR spectra, UV-vis spectrum, TEM and fluorescent spectrum of NIR-1

**Fig. 2:**
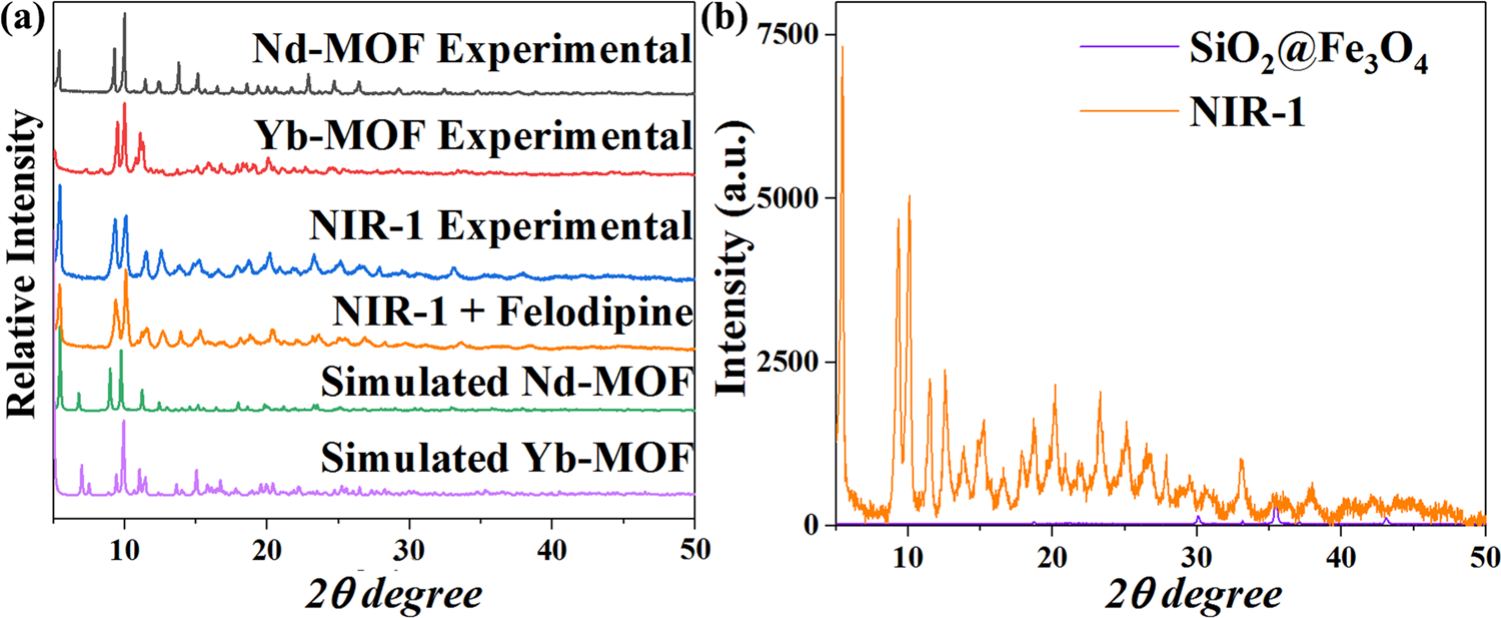
PXRD characterizations of NIR-1. **a** PXRD of Yb-MOF, Nd-MOF and **NIR-1**. **b** PXRD intensity of SiO_2_@Fe_3_O_4_ and **NIR-1**.

Powder X-ray Diffraction (PXRD) patterns of Yb-MOF, Nd-MOF and **NIR-1** have been characterized, all of them possess the similar structural motifs, which also confirm the purity of **NIR-1** (Fig. 2a). Additionally PXRD intensity of SiO_2_@Fe_3_O_4_ as the magnetic core of **NIR-1** is very weak, which is consistent with previous reported work (Fig. 2b)^20^. Single-crystal structure of Yb-MOF and Nd-MOF were described in detail in a previous report (Supplementary Fig. 1 and Supplementary Fig. 2)^25^. In Yb-MOF, L^3-^ is linked through two central Yb atoms, Yb1 and Yb2, in which all the eight O atoms coordinate the central Yb atoms. Yb1 is coordinated by five O atoms (O1, O2, O4, O6, O8) from carboxyl groups of L^3-^ and three O atoms (O3, O5, O7) from H_2_O, Yb2 is coordinated by seven O atoms (O1, O2, O3, O5, O6, O7, O8) from carboxyl groups of L^3-^ and one atom O (O4) from DMF (Supplementary Fig. 1a). In the Nd-MOF, one L^3-^ coordinated with three Yb atoms (Nd1), which have the same coordination environment. Nd1 is coordinated by seven O atoms (O1, O2, O5, O6, O7, O8, O9) from carboxyl groups of L^3-^ and two O atoms (O3, O4) from DMF (Supplementary Fig. 2a).

In the Ultraviolet-visible (UV-vis) spectrum, **NIR-1** has two absorption peaks, and the maximum absorption occurs at 330 nm, which can be attributed to the H_3_L ligand (Supplementary Fig. 3a). Felodipine has two absorption peaks, located at 255 nm and 355 nm (Supplementary Fig. 3b). In the Fourier Transform Infrared (FT-IR) spectra (Supplementary Fig. 4a), the stretching vibration peak of the C = O bond in **NIR-1** is 1660 cm^-1^, which is shifted to lower wavenumber compare with the stretching vibration peak of the C = O bond in the pure ligand (1688 cm^-1^), demonstrating the carboxyl groups are coordinated with Yb^3+^ and Nd^3+^^26–34^. Fluorescent properties of **NIR-1** in the DMF solution are also investigated (Supplementary Fig. 4b). **NIR-1** has three NIR characteristic emission peaks at 978 nm (^2^F_5/2_ → ^2^F_7/2_) origin from Yb^3+^ and 1056 nm (^4^F_3/2_ → ^4^I_11/2_) origin from Nd^3+^, respectively. The excitation spectrum of **NIR-1** indicated that 330 nm excitation light source is the most suitable experimental conditions (Supplementary Fig. 5). The quantum yield of **NIR-1** is 0.07%. We think that the low quantum yield are mainly influenced by the following factors: Firstly, **NIR-1** has a layered structure, the main contribution to quantum yield comes from Yb/Nd ions emissions in the outermost layer and most of the Yb/Nd ions are in the interior of **NIR-1**; Secondly, we only tested the quantum yield from 950 to 1100 nm (980 nm for Yb and 1056 nm for Nd) and did not include the ligand emission.

**Fig. 3:**
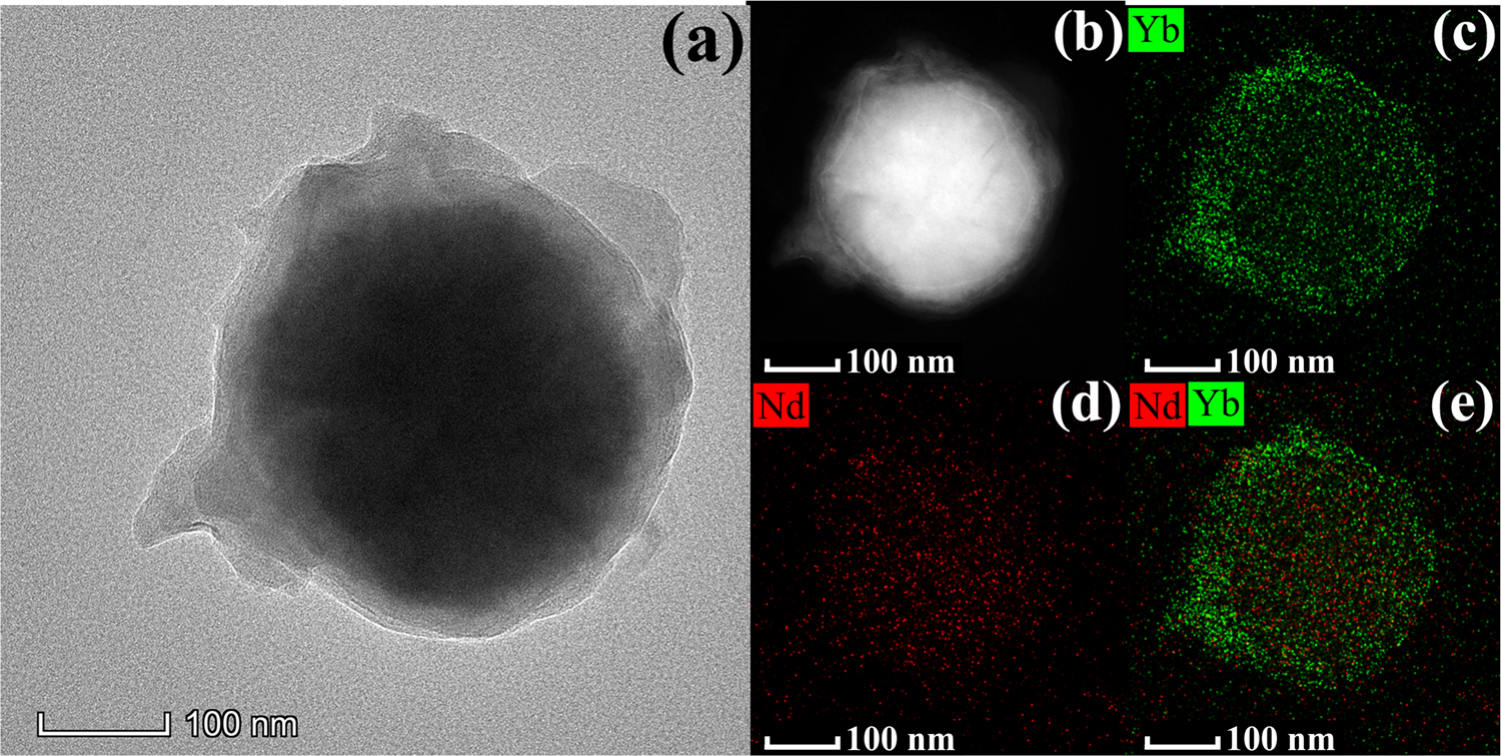
TEM characterizations of NIR-1. **a** TEM image of **NIR-1**. **b**–**e** TEM mapping images of **NIR-1**.

**Fig. 4:**
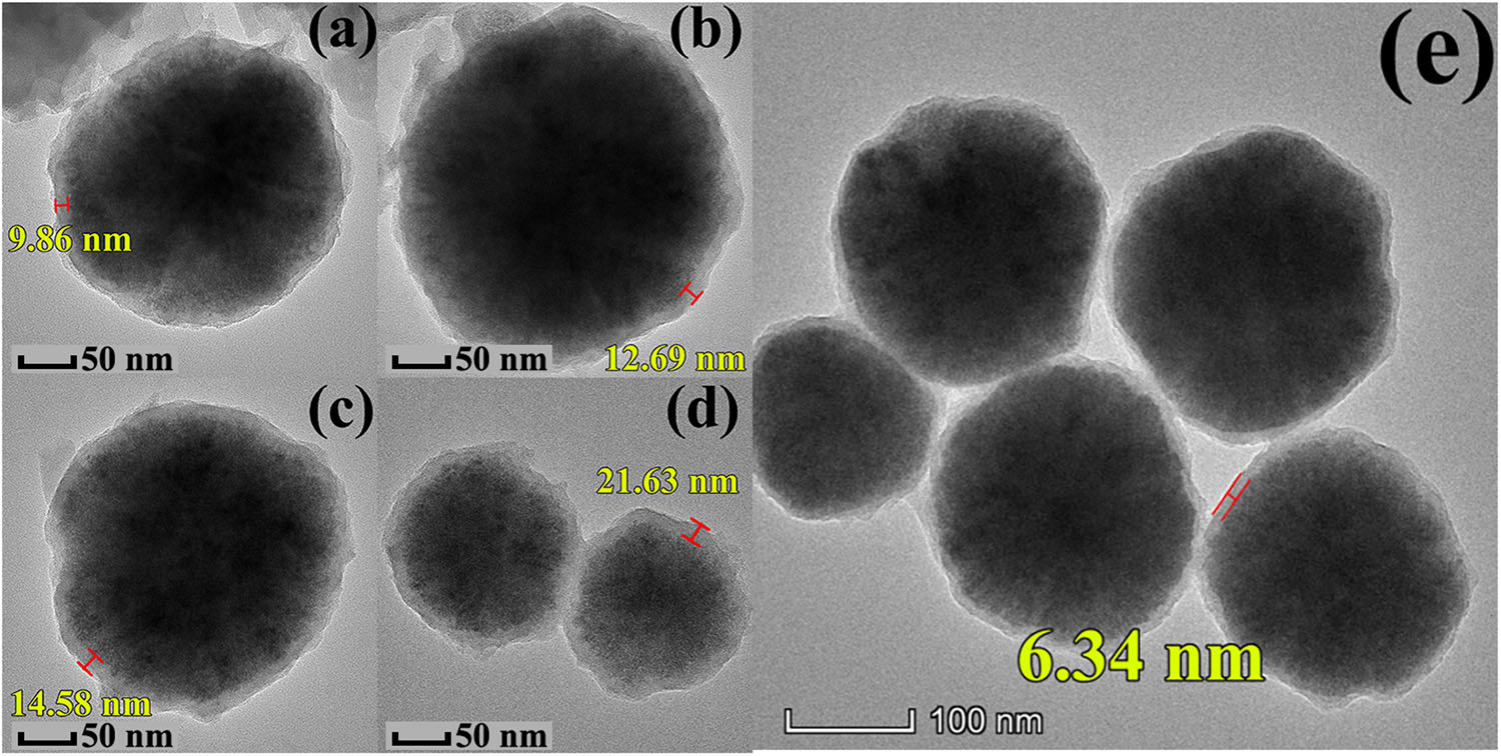
TEM characterizations of different NIR-1. **NIR-1** particle of 3 cycles (**a**), 7 cycles (**b**), 14 cycles (**c**) and 21 cycles (**d**). **e** TEM image of Fe_3_O_4_@SiO_2_.

In Transmission Electron Microscope (TEM) images, the mapping images showed the multilayer structure of **NIR-1** (Fig. 3)^35–40^. The magnetic core is inside, which consist of magnetic iron oxide and silicon dioxide. The outer shell layer structure is composed of Yb-MOF and Nd-MOF respectively (Fig. 3c–e). Yb-MOF and Nd-MOF are uniformly distributed on the surface of the magnetic core. Elements content of Yb and Nd is 0.31% and 0.08%, respectively (Supplementary Table 1). The thickness of the MOF layer can be controlled the by layer-by-layer method. Figure 4 shows that the outer layer thickness of **NIR-1** increases as the number of layer-by-layer methods increases (9.86 nm to 21.63 nm). As the pure Fe_3_O_4_@SiO_2_ magnetic core are only coated with SiO_2_, the thickness of outer layer is <6.5 nm (Fig. 4e).

### Variable temperature solid-state fluorescent spectrum of NIR-1

**Fig. 5:**
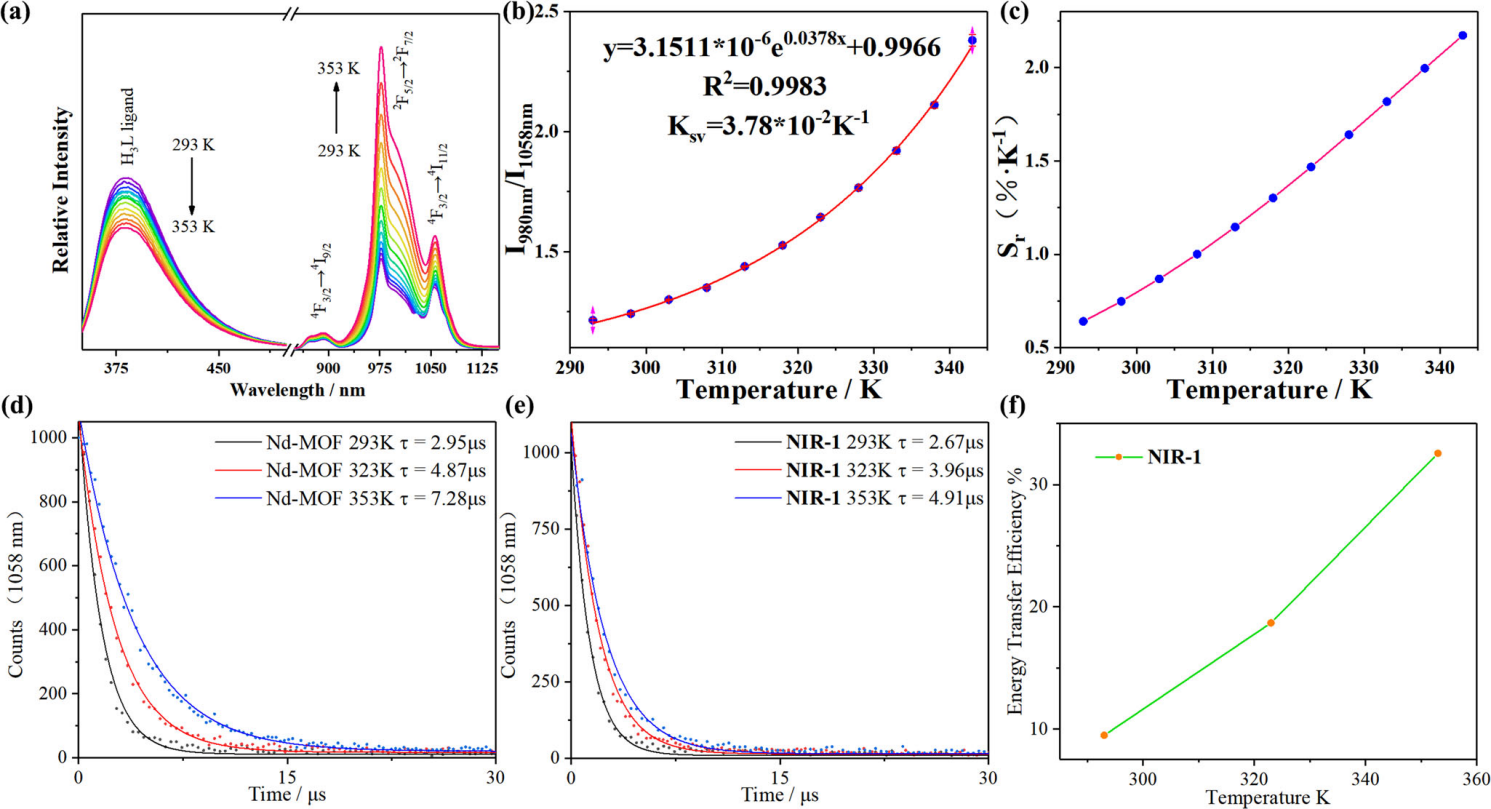
Solid-state Luminescent characterizations of NIR-1. **a** Variable temperature solid fluorescent spectra of **NIR-1** varied from 293 K to 353 K. **b** Non-linear relationship between fluorescent intensity ratio of Yb^3+^ (980 nm) and Nd^3+^ (1058 nm) and temperature. **c** Relative sensitivity (Sr) for **NIR-1** in different temperature. **d** The solid luminescent lifetime of Nd-MOF at the different temperature at 1058 nm which excited at 330 nm. **e** The solid luminescent lifetime of **NIR-1** at the different temperature at 1058 nm which excited at 330 nm. **f** Temperature dependence of the energy transfer efficiency from Nd^3+^ to Yb^3+^ in **NIR-1**.

In this work, solid-state photo-luminescence spectroscopy of **NIR-1** in the temperature range of 293 K to 353 K has been observed (Fig. 5a). With the temperature increase, the intensities of both emission bands gradually increase, the luminescence intensity of Yb^3+^ increases much more obviously than that of Nd^3+^. Based on the variation difference between these two emissions, the two emissions located at 980 nm and 1058 nm can be selected as ratiometric temperature sensors, which can be applied as self-calibrating temperature measurements. To simplify the description, the ratio of dual emission at 980 nm and 1058 nm represents Δ. On this basis, the temperature sensing performance of **NIR-1** was quantitatively described. It is a cure with an increasing trend and shows a good nonlinear relationship between Δ and temperature (Fig. 5b, c). The nonlinear formula is fitted to equation 1: Δ = 3.1511*10^-6^e^0.0378T^ + 0.9966, and correction coefficient is 0.9983. S_r_ (relative thermal sensitivity) can be calculated by the equation |∂Δ/∂T | /Δ. The definition of |∂Δ/∂T | /Δ is signal to noise ratio of the luminescent spectrometer which is 30000:1. According to the nonlinear equation 1, the evolution of S_r_ under different temperature was presented, the maximum S_r_ for **NIR-1** is 2.17% K^-1^ at 343 K. To verify the stability of the **NIR-1**, we carried out repeated experiments. Through the cyclable experiments (Supplementary Fig. 10), **NIR-1** can be reusable at least 3 times for temperature sensing which reflects the stability of **NIR-1**.

Host-guest energy transfer process is affected by temperature change, which leads to the increase of emission intensity. To confirm the energy transfer direction, solid-state **NIR-1** has three characteristic emission bands at 890 nm, 980 nm and 1058 nm, which represent the emission of Nd^3+^ (^4^F_3/2_ → ^4^I_9/2_), Yb^3+^ (^2^F_5/2_ → ^2^F_7/2_) and Nd (^4^F_3/2_ → ^4^I_11/2_) (Fig. 5a), which means that the energy transfer direction should be from the Nd^3+^ to Yb^3+^ in **NIR-1**. Additionally, there is overlap between emission spectrum of L^3-^ and excitation spectrum of Yb^3+^/Nd^3+^ (Supplementary Fig. 5), which represents to there is a strong energy transfer between ligand and lanthanide ions. The reason why enhancement of temperature with the increase emission of **NIR-1** based on a thermally driven phonon-assisted transfer mechanism. The similar situation can be found in previous work^41^, when the temperature increases from 90 K to 240 K, the lifetime of Tb-BABDC-PBMA (^5^D_4_) decreases by 49.5%, while the lifetime of Eu_0.0025_Tb_0.9975_-BABDC-PBMA (^5^D_4_) decreases by 81.3%. This result indicates that the energy transfer efficiency of Tb^3+^ to Eu^3+^ is stably enhanced at high temperature. Similarly, the energy transfer efficiency of Nd^3+^ to Yb^3+^ is also stably enhanced at high temperature.

To explore the relationship between energy transfer efficiency and temperature, the solid luminescent lifetime of **NIR-1** and Nd-MOF at 1058 nm was investigated, respectively. With the temperature increasing (293 K to 353 K), the lifetime of **NIR-1** increased from 2.67 μs to 4.91 μs, while the lifetime of Nd-MOF inreased from 2.95 μs to 7.28 μs (Figs. 5d and 5e). Obviously, the presence of energy transfer acceptor Yb^3+^ in **NIR-1** leads to the difference of Nd^3+^ emission lifetime parametera between **NIR-1** and Nd-MOF. The efficiency of energy transfer between the donor (Nd^3+^) and acceptor (Yb^3+^) can be calculated from the lifetime of donor luminescence: E = 1-*τ*_da_/*τ*_d_, where *τ*_da_ and *τ*_d_ are the donor’s excited-state lifetime in the presence and absence of the acceptor, respectively. As shown in Fig. 5f, the energy transfer efficiency from Nd^3+^ to Yb^3+^ is gradually increases with the temperature increasing, which proved that the phonon-assisted Foster transfer mechanism^42–49^.

Compared with other existing dual emission MOFs-based thermometers^50–56^, S_r_ value of **NIR-1** is higher (Table 1). The results indicated that **NIR-1** has the excellent fluorescent temperature sensing performance in the near-infrared emission region.

**Table 1:** Comparison of maximum S_r_ values about different materials.

Materials	Max. Sr[% K^-1^]	Ref.
**NIR-1**	2.17 (343K)	This work
Tb_0.93_Eu_0.07_-BODSDC	0.23 (300K)	^50^
CQDs@UiO-66-(COOH)_2_	1.3 (297K)	^51^
Tb_0.8_Eu_0.2_BPDA	1.19 (313K)	^52^
Gd_0.84_Er_0.01_Yb_0.15_BiW_2_O_9_	1.74 (303K)	^53^
Ln(ad)_0.5_(phth)-(H_2_O)_2_	1.21 (303K)	^54^
Eu_0.05_Tb_1.95_-PDC	1.37 (333K)	^55^
THA@Eu-NMOF@Fe/TA	0.59 (333K)	^56^

### Ratiometric luminescent sensor for felodipine by NIR-1

Insufficient use of hypertension drugs can cause a rebound in blood pressure, while overdose can cause liver damage, hypotension, and even drug poisoning^57^. Therefore, it is very significant to establish highly sensitive and efficient methods for qualitative and quantitative detection of hypertension drugs. In this work, we pioneered the detection of felodipine by using **NIR-1**.

**Fig. 6:**
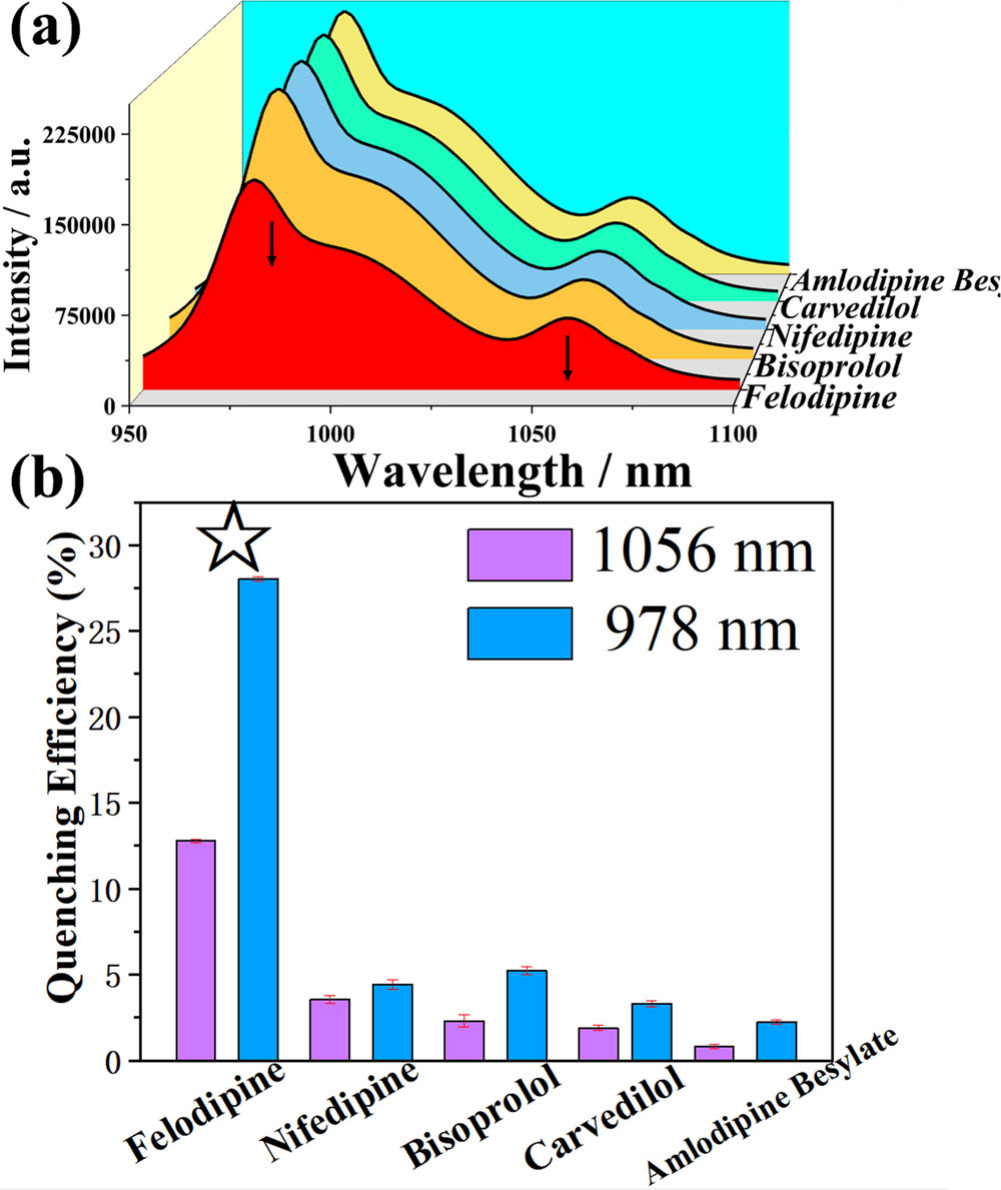
Selectivity experiments of NIR-1. **a** Luminescent spectra of **NIR-1** suspension (0.2 g/L, 1 mL) by the addition of different drugs solutions (40 nM) when excited at 330 nm. **b** Quenching efficiency (978 nm and 1056 nm) when different drugs solutions (40 nM) were added into **NIR-1** suspensions (0.2 g/L), respectively (exciting position is 330 nm). The error bars are the standard deviation of three parallel experiments.

**NIR-1** is a near-infrared luminescent probe used to sense various hypertension drugs. We prepared five drugs for the treatment of hypertension (felodipine, nifedipine, bisprolol, carvedilol, and amlodipine besylate) to test the luminescent sensing properties of **NIR-1**. The emission peaks located at 978 nm and 1056 nm were quenched to different degrees with the addition of drugs (40 nM). It is worth noting that the quenching amount of luminescent intensity caused by the addition of felodipine is the largest compared with other medications. The quenching efficiency at the same concentration is 4-10 times that of other drugs. Obviously, this difference in quenching efficiency reveals that felodipine can be selectively identified among many antihypertensive drugs (Fig. 6).

**Fig. 7:**
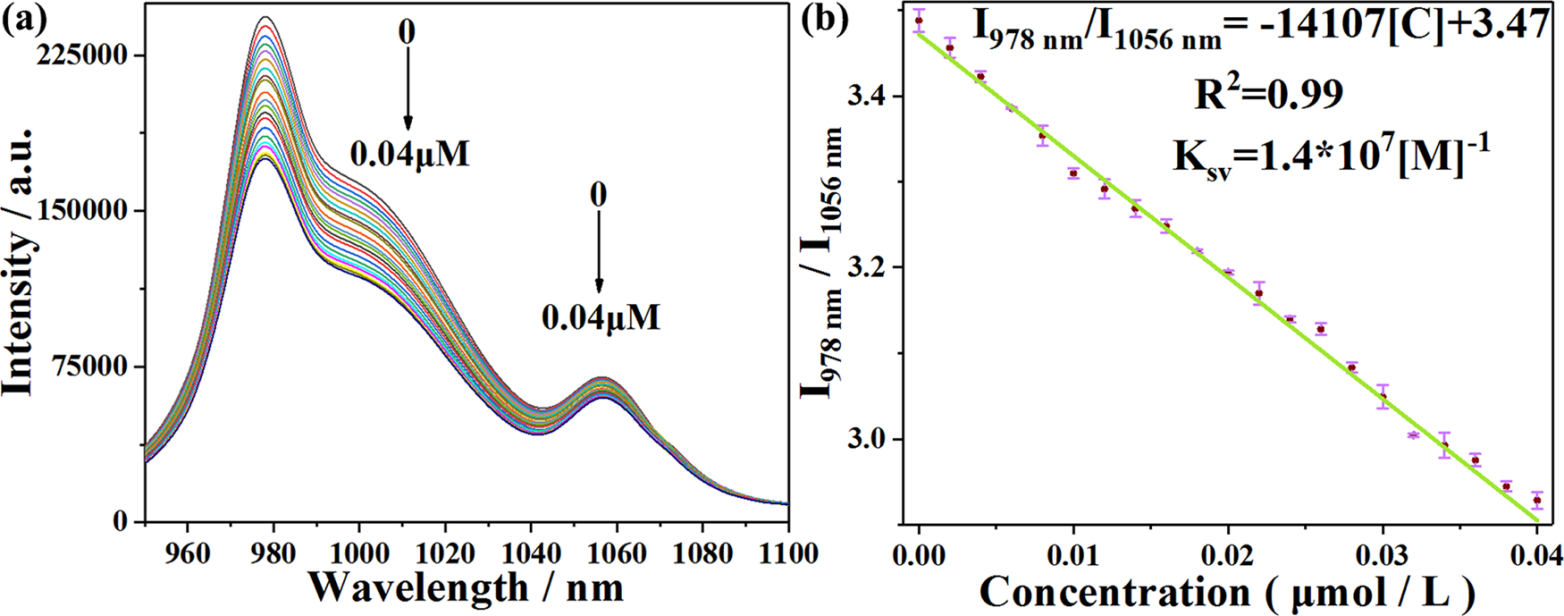
Quantitative detection experiments of NIR-1. **a** Luminescent spectra of **NIR-1** (0.2 g/L 14 cycles) with the addition of felodipine in different concentration (0-40 nM) when excited at 330 nm. **b** Linear relationship between fluorescent intensity ratio of **NIR-1** (I_978 nm_/I_1056 nm_) and the concentration of felodipine. The error bars are the standard deviation of three parallel experiments.

To further explore the potential of **NIR-1** in felodipine quantitative detection, the suspension of **NIR-1** was titrated with felodipine. The emission peaks at 978 nm and 1056 nm were gradually quenched with the addition of the felodipine (from 0-40 nM) (Fig. 7a). To explore the relationship between the concentration of felodipine and luminescent intensity ratio of **NIR-1**, which are represented by I_978 nm_/I_1056 nm_ and [C] (concentration of felodipine). I_978 nm_/I_1056 nm_ and [C] has a good linear relationship using the following equation: I_978 nm_/I_1056 nm_ = -14107 [C] + 3.47 (*R*^2^ = 0.99), the quenching effect (K_sv_) is calculated to be 1.4 × 10^7^ [M]^-1^, and low of detection (LOD) is 6.39 nM (Fig. 7b).

In addition, **NIR-1** in prepared using different synthesis steps (7 cycle steps and 21 cycle steps) to conduct felodipine detection experiments. For the **NIR-1** material obtained by 7 cycle steps, the relationship between the concentration of felodipine and I_978 nm_/I_1056 nm_ is calculated as follows: I_978 nm_/I_1056 nm_ = -6968 [C] + 3.20 (*R*^2^ = 0.98), the K_sv_ value is calculated to be 7.0×10^6^ [M]^-1^ (Supplementary Fig. 6); For the **NIR-1** obtained by 21 cycle steps, the relationship between the concentration of felodipine and I_978 nm_/I_1056 nm_ is calculated as follows: I_978 nm_/I_1056 nm_ = -12486 [C] + 5.28 (*R*^2^ = 0.99), the K_sv_ is calculated to be 1.2 × 10^7^ [M]^-1^ (Supplementary Fig. 7). Both K_sv_ values of **NIR-1** nanoballs synthesized by different cycle steps are smaller than the K_sv_ **of NIR-1** obtained by 14 cycle steps. This indicated that **NIR-1** obtained by 14 cycle steps has the best performance in felodipine detection, which may attributed to **NIR-1** obtained by 14 cycles possess Ln^3+^ strong emission and appropriate number of defect sites to attract felodipine further to realize quenching effect.

To highlight the advantages and stability of **NIR-1**, the parallel detection experiment of felodipine based on Yb-MOF and Nd-MOF is also performed. For Yb-MOF (Supplementary Fig. 8), the emission peaks at 975 nm was gradually quenched with the addition of felodipine in different concentration (from 0-40 nM). The following equation is used: I_0_/I = 11433[C] + 0.0076 (*R*^2^ = 0.98, K_sv_ = 1.1 × 10^7^ [M]^-1^); For Nd-MOF (Supplementary Fig. 9), the emission peaks at 1058 nm was gradually quenched with the addition of felodipine in different concentration (from 0-40 nM). The following equation is used: I_0_/I = 8438[C] + 0.0094 (*R*^2^ = 0.96, K_sv_ = 8.4 × 10^6^ [M]^-1^). Compared with Yb-MOF and Nd-MOF, **NIR-1** has a higher value of K_sv_, additionally the ratiomeric probe of **NIR-1** can more efficiently avoid the external interference.

### Anti-interference performance of NIR-1 in the felodipine detection process

The antihypertensive drugs used in this work are divided into two types: Calcium channel antagonists (felodipine, nifedipine, and amlodipine besylate) and beta-blocker (bisoprolol and carvedilol). Doctors usually combine different antihypertensive drugs for patients with severe hypertension to achieve better treatment effects. Therefore, in felodipine’s detection process, other medicines often interfere. Consequently, it is necessary to conduct selective testing of mixed drugs for **NIR-1**. We have prepared four additional samples: A mixture of felodipine and carvedilol, a mixture of felodipine and bisoprolol, a mixture of carvedilol and bisoprolol, a mixture of felodipine, bisoprolol, and carvedilol. Each drug in the solutions was a concentration of 2 μM. Suspension of **NIR-1** is titrated by using mixed durg solutions. It can be concluded that felodipine plays a leading role in the quenching effect to **NIR-1** emission, the contribution of bisoprolol and carvedilol to luminescent quenching is not apparent, but with the addition drug types, the quenching efficiency also slightly increased (1.4 × 10^7^ [M]^-1^ to 1.6 × 10^7^ [M]^-1^) (Fig. 7b and Supplementary Fig. 11). This makes it is possible to judge the sample contains additional drugs by the value of quenching efficiency. To further explore the **NIR-1**’s quantitative detection ability of felodipine in the presence of interference, the suspension of **NIR-1** was titrated with the mixture of calcium channel antagonists drugs (Supplementary Fig. 11a). The relationship between concentration of the mixture and I_978 nm_/I_1056 nm_ is calculated as follows: I_978 nm_/I_1056 nm_ = -15980[C] + 3.36 (*R*^2^ = 0.99), the K_sv_ is calculated to be 1.6 × 10^7^ [M]^-1^ (Supplementary Fig. 11b)^53^. This reflects the **NIR-1** has the excellent anti-interference ability in the sensing experiment.

### Blind box experiment for the analysis of background drug interference

**NIR-1** not only has excellent anti-interference performance, but also has the ability to analyze background interference (judge the sample contains additional drugs by the value of quenching efficiency). For the five drug solutions with known concentration, the quenching efficiency increased slightly with the increase number of drugs (Supplementary Fig. 12a). Then, we conducted a blind box experiment, firstly, unlabled A is  containing two solutions: felodipine solution, a mixed solution containing felodipine, bisoprolol, and carvedilol. Each drug in the solutions was the same concentration. The type and concentration of the solutions in the sample B. Sample B contain two solutions and further detected by **NIR-1**. The experimental results show that the two solutions behave differents quenching efficiency to **NIR-1**. The solution with higher quenching efficiency contained three drug components than pure felodipine solution towards **NIR-1**, it is consistent with the previous experimental results (Supplementary Fig. 12b).

### Detection application of NIR-1 for felodipine in the real biological samples

Further, the detection application of **NIR-1** for felodipine in the real serum samples is also performed. The maximum blood concentration(C_max_) is an important index to evaluate the effect of drug therapy, when the connection of felodipine is higher than C_max_, patients will be suffer the danger even damage their life., To help patients obtain the better therapeutic effect, the development of facile and sensitive felodipine detection platform in the range of C_max_ is of great significance. Therefore, **NIR-1**’s luminescent performance towards felodipine is performed in the human serum. Based on previous studies, the C_max_ of felodipine is 12 nM^58–61^. Felodipine solution (in human serum) is used to titrate **NIR-1**. The recovery rate can be found between 98.69%-102.53% and the RSD is <1.14% (Table 2). This demonstrates the **NIR-1** has excellent performance to detect felodipine in human serum.

**Table 2:** Analytical results for the detection of felodipine in human serum.

Samples	Added (nM)	Found (nM)	Recovery (%)	RSD (*n* = 3, %)
Sample-1	0	Not detected		
Sample-2	7.50	7.69	102.53	0.45
Sample-3	12.50	12.46	99.68	1.14
Sample-4	17.50	17.27	98.69	0.16
Sample-5	22.50	22.92	101.87	0.72

### Detection mechanism of felodipine based on NIR-1 sensing platform

**Fig. 8:**
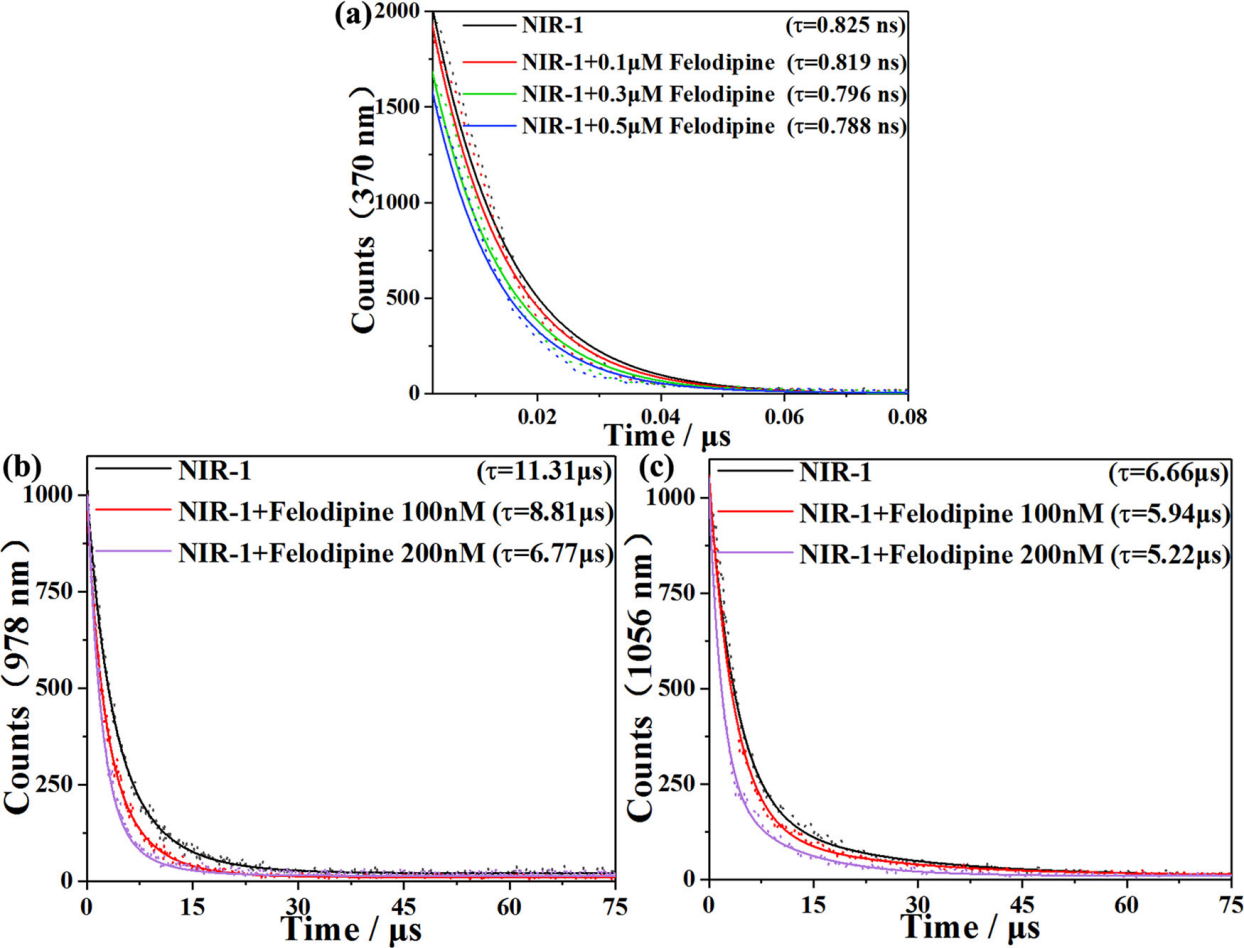
Luminescent lifetime characterizations of NIR-1. **a** Luminescent lifetime of the H_3_L ligand with the addition of felodipine in different concentration (from 0-500 nM) at 370 nm, which excited at 330 nm. **b** Luminescent lifetime of the **NIR-1** with the addition of felodipine in different concentration (from 0-200 nM) at 978 nm, which excited at 330 nm. **c** Luminescent lifetime of the **NIR-1** with the addition of felodipine in different concentration (from 0-200 nM) at 1056 nm, which excited at 330 nm.

To explore the luminescent quenching mechanism for felodipine based on **NIR-1** sensing platform, PXRD and luminescent lifetime of **NIR-1** in the detection process is investigated. Firstly, PXRD result showed that **NIR-1** could keep its structural stability in the detection process of felodipine, luminescent quenching of **NIR-1** is not caused by structural collapse^62^. Secondly, luminescent lifetime parameters are significant for analyzing the detection mechanism. The lifetime of **NIR-1** solution (0.2 g/L) with the addition of felodipine is investigated. The lifetime of L^3-^ at the position of 370 nm has been decreased from 0.825 ns to 0.788 ns with the addition of felodipine in different concentration (from 0 to 500 nM) (Fig. 8a) (Table 3). Given to there are some aromatic rings in felodipine so it can attach with H_3_L through the π-π stacking and oxygen atom and “-NH” group in felodipine can easily combine with H_3_L in **NIR-1** through hydrogen bonding interaction, these excited-state hydrogen bond/ π-π stacking interactions strengthening facilitates internal conversion, which lead to felodipine can interrupt the energy transfer progress from H_3_L to Ln^3+^ ions^63^. The NIR lifetime of Yb-MOF at the position of 978 nm has been reduced from 11.31 μs to 6.77 μs (Fig. 8b) (Table 4) and the NIR lifetime of Nd-MOF at the position of 1056 nm has been reduced from 6.66 μs to 5.22 μs with the addition of felodipine in different concentration (from 0-200 nM) (Fig. 8c) (Table 5), which means that felodipine can further lead to energy transfer process between Yb^3+^ and Nd^3+^, which leads to the emission quenching of the Yb^3+^/Nd^3+^^64,65^. As a result, the quenching effect of Yb^3+^/Nd^3+^ emissions bands were mainly caused by the hydrogen bond/π-π stacking interactions and interruption of energy transfer between Yb^3+^ and Nd^3+^ is caused by felodipine^66,67^.

**Table 3:** Luminescent lifetime parameters of the H_3_L ligand in the felodipine detection process.

Name	Testing sample	Average lifetime (ns)	T_1_ (ns)	B_1_(%)	T_2_ (ns)	B_2_(%)
**NIR-1** (λ_em_ = 370 nm)	No	0.8258	0.4933	88.84	3.4724	11.16
**NIR-1** (λ_em_ = 370 nm)	Felodipine 100 nM	0.8190	0.4859	86.87	3.0254	13.13
**NIR-1** (λ_em_ = 370 nm)	Felodipine 200 nM	0.8106	0.5052	88.51	3.1630	11.49
**NIR-1** (λ_em_ = 370 nm)	Felodipine 500 nM	0.7881	0.4893	86.29	2.6682	13.71

**Table 4:** Luminescent lifetime parameters of the **NIR-1** 978 nm emission in the felodipine detection process.

Name	Testing sample	Average lifetime (μs)	T_1_ (μs)	B_1_(%)	T_2_ (μs)	B_2_(%)
**NIR-1** (λ_em_ = 978 nm)	No	11.3111	5.9054	35.98	14.3493	64.02
**NIR-1** (λ_em_ = 978 nm)	Felodipine 100 nM	8.8110	4.0723	32.59	11.1019	67.41
**NIR-1** (λ_em_ = 978 nm)	Felodipine 200 nM	6.7703	3.7737	60.57	11.3736	39.43

**Table 5:** Luminescent lifetime parameters of the **NIR-1** 1056 nm emission in the felodipine detection process.

Name	Testing sample	Average lifetime (μs)	T_1_ (μs)	B_1_(%)	T_2_ (μs)	B_2_(%)
**NIR-1** (λ_em_ = 1056 nm)	No	6.6630	1.8413	39.71	9.8388	60.29
**NIR-1** (λ_em_ = 1056 nm)	Felodipine 100 nM	5.9413	1.9611	50.34	9.9761	49.66
**NIR-1** (λ_em_ = 1056 nm)	Felodipine 200 nM	5.2170	1.7662	55.86	9.5841	44.14

## Discussion

In this work, multi-emission ratiometric luminescent probe **NIR-1** was synthesized by the LBL deposition method, which has been characterized by PXRD, FT-IR, UV-vis spectrum, TEM and luminescent spectrum. **NIR-1** achieves temperature sensing in the range of 293 K to 353 K based on the emission intensity ratio of Yb^3+^ (978 nm) and Nd^3+^ (1056 nm). In addition, **NIR-1** also can be used as a ratiometric luminescent sensor for felodipine detection with high efficiency and sensitivity. We believe that NIR hierarchical Ln-MOF has great potential application in drugs detection, the detection of C_max_ in the human serum sample should help patients to avoid drug abuse. NIR-materials probes can avoid many external interference such as the emission located between 400-600 nm from organism, As a result, it can be expected that NIR hierarchical Lanthanide-MOF magnetic core-shell nanoparticles would have great potential applications to other molecular drugs' detection in real body fluid.

## Experimental section

### General methods

H_3_L (Terphenyl-3,4'',5-tricarboxylic acid) was purchased from JiNan HengHua Technology Co. Ltd. SiO_2_@Fe_3_O_4_ magnetic particle was purchased from BioMag beads Co. Ltd. The chemicals used in this word were obtained from commercial sources and can be used without further purification. Powder X-Ray Diffraction (PXRD) was characterized by a D/Max-2500 X-ray diffractometer using Cu-Kα radiation. FT-IR spectra (4000-500 cm^-1^) were recorded by using a NICOLET 6700 FT-IR spectroscope with KBr pellets (NICOLET, USA). Ultrasonic preparation was carried by the SB-100DT Ultrasonic bath (XJ Biotechnology Instrument, Zhe Jiang, China). PerkinElmer Lambda 35 spectrophotometer was used to determine ultraviolet-visible (UV-vis) adsorption spectra. PL emission spectra were recorded on an RF-5301 spectrophotometer (Daojin, Japan). PL lifetime tests were performed by Fluorolog-3, Horiba Jobin Yvon (USA) and FLS 980. The morphology and size of Nd-MOF@Yb-MOF@SiO_2_@Fe_3_O_4_ were characterized by Tecnai G^2^ F20 Transmission Electron Microscope (TEM; FEI instrument, USA).

### Solvothermal synthesis of Yb-MOF and Nd-MOF

Yb-MOF was synthesized by reported work^25^. Typically, a mixture of Ytterbium(III) acetate (0.0211 g, 0.05 mmol) and H_3_L (0.0161 g, 0.05 mmol) was added to 6 mL the mixture of DMF/H_2_O (v/v, 4/2) solution, stir well at room temperature. Then the mixture was added to a 25 ml Teflon-lined steel autoclave and heated to 393 K. Kept at this temperature for 72 h. Then the resulting mixture was slowly cooled to room temperature. The product was washed with DMF and H_2_O three times to obtain colorless crystal powder. Yield: 55% (based on the H_3_L ligand).

The synthesis method of Nd-MOF was the same as that of Yb-MOF just usage Neodymium(III) trichloride (0.0125 g, 0.05 mmol) not Ytterbium(III) acetate. The product was washed with DMF and H_2_O three times to obtain colorless crystal powder. Yield: 58% (based on the H_3_L ligand).

### Synthesis of Nd-MOF@Yb-MOF@SiO_2_@Fe_3_O_4_ (NIR-1)

Firstly, 1 mL SiO_2_@Fe_3_O_4_ magnetic particle suspension (0.5 g/mL) was added to a 25 mL beaker. Step 1: 5 mL Ytterbium(III) acetate solution (8 mM, DMF and H_2_O as solvent) was added to the beaker. The mixture was heated to 353 K and stirred for 5 min. After washing and centrifuging (8000 r/min, 3 min), the product was transferred to a beaker, and the supernatant was removed. Step 2: Step 2 was the same as that of step 1 just usage 5 mL H_3_L ligand suspension (5 mM, DMF and H_2_O as solvent) not Ytterbium(III) acetate solution. Step 3: Step 3 was the same as that of step 1 just usage 5 mL Neodymium(III) trichloride solution (2.5 mM, DMF and H_2_O as solvent) not Ytterbium(III) acetate solution. Step 4: Same as step 2. Step 1–4 forms a complete cycle of steps, the above synthetic routes can repeat the cycle for 14 times. The white powdered **NIR-1** was obtained after washed with DMF and ethanol three times, respectively.

### Preparation of different analytical solutions

#### Preparation of Yb-MOF suspension

Yb-MOF (2 mg) and DMF (10 mL) were added to a 25 mL beaker, then the mixture was under an ultrasonic bath 30 min to prepare the 0.2 g/L Yb-MOF suspensions.

#### Preparation of Nd-MOF suspension

Nd-MOF (2 mg) and DMF (10 mL) were added to a 25 mL beaker, then the mixture was under an ultrasonic bath 30 min to prepare the 0.2 g/L Nd-MOF suspensions.

#### Preparation of Nd-MOF@Yb-MOF@SiO_2_@Fe_3_O_4_ (NIR-1) suspension

**NIR-1** (2 mg) and DMF (10 mL) were added to a 25 mL beaker, then the mixture was under ultrasonic bath 30 min to prepare the 0.2 g/L **NIR-1** suspensions.

#### Preparation of different drug solutions

The following drugs such as felodipine, Nifedipine, Bisoprolol, Carvedilol, Amlodipine Besylate were added to 10 mL DMSO under an ultrasonic bath for 5 min to prepare the 2 μM drug solutions for future use.

#### Preparation of biological samples

Felodipine (3.8 mg) were added to 10 mL DMSO under an ultrasonic bath for 5 min to prepare the 1 mM felodipine solutions. Then, dilute the felodipine solution with serum to 2 μM for future use.

## Supplementary information

Supplementary Information

## Data Availability

The authors declare that all data in this work are available within the article and supplementary information files and from the corresponding author on request.
