# Supplementary material for: Near-infrared magnetic core-shell nanoparticles based on lanthanide metal-organic frameworks as a ratiometric felodipine sensing platform
Source: Commun Chem. 2023 May 18;6:96. doi: 10.1038/s42004-023-00893-7 (PMC10195807; doi:10.1038/s42004-023-00893-7)
Supplement: Supplementary file 1 — Supplementary Information [file 42004_2023_893_MOESM1_ESM.pdf]

Supplementary information

Near-infrared magnetic core-shell nanoparticles based on lanthanide metal-organic frameworks as a ratiometric felodipine sensing platform

Yu-Peng Jiang[a], Xin-Hui Fang[a], Qian Wang[a], Jian-Zhong Huo[a], Yuan-Yuan Liu[a], Xin-Rui Wang[a*] and Bin Ding[a*]

[a]*Tianjin Key Laboratory of Structure and Performance for Functional Molecule, College of Chemistry, Tianjin Normal University, 393 Binshui West Road, Tianjin 300387, PR China*

**To whom correspondence should be addressed.*

E-mail:wangxinrui-tjnu@outlook.com; hxxydb@mail.tjnu.edu.cn *Received Date (automatically inserted by the publisher)*

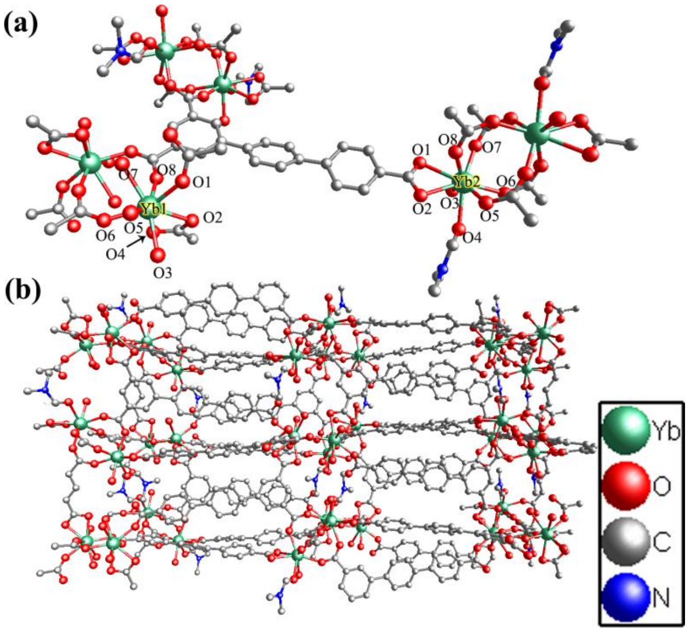

Supplementary Fig. 1 (a) Coordination mode of Yb in Yb-MOF and (b) 3D framework structure of Yb-MOF.

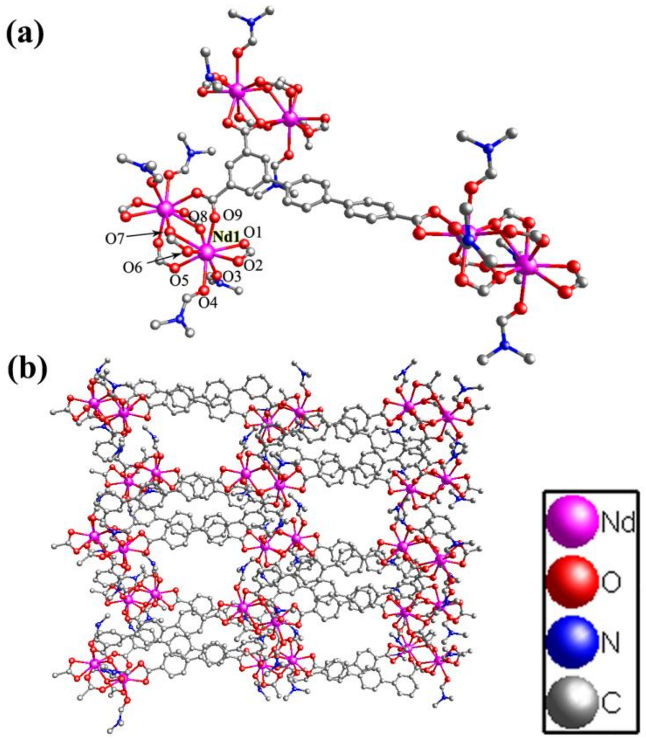

Supplementary Fig. 2 (a) Coordination mode of Nd in Nd-MOF and (b) 3D framework structure of Nd-MOF.

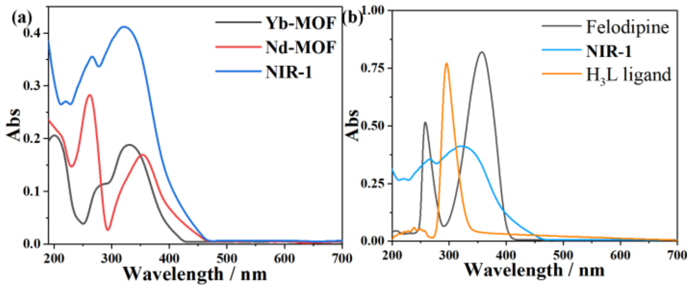

Supplementary Fig. 3 (a) UV-vis spectra of Yb-MOF, Nd-MOF and **NIR-1** in DMF solutions; (b) UV-vis spectra of felodipine, **NIR-1** and H_3L ligand.

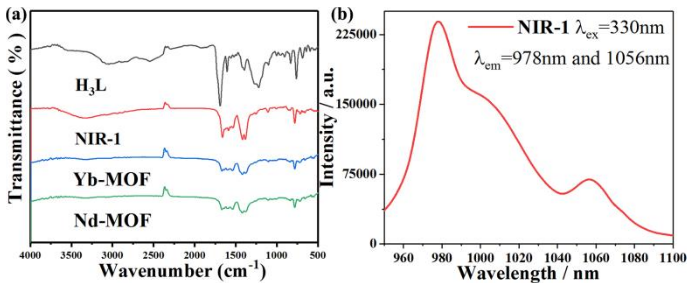

Supplementary Fig. 4 (a) FT-IR spectra of Nd-MOF, Yb-MOF, H_3L and **NIR-1**; (b) Fluorescent emission spectra of the **NIR-1** in DMF solution, which excited at 330 nm and slit is 12 nm.

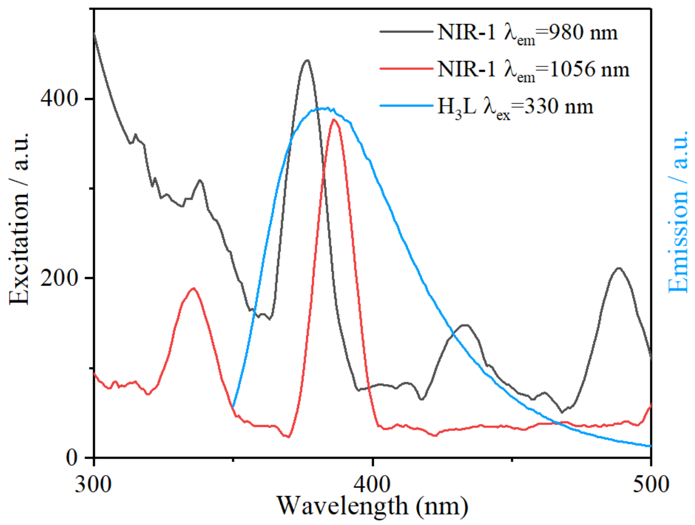

Supplementary Fig. 5 Excitation spectra of **NIR-1** and emission of H_3L.

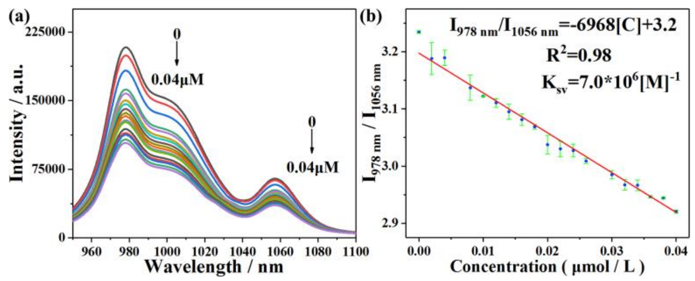

Supplementary Fig. 6 (a) Luminescent spectra of the **NIR-1** (0.2 g/L, 7 cycles) with the addition of felodipine in different concentration (0-40 nM) when excited at 330 nm, the slit is 12 nm; (b) The linear relationship between fluorescent intensity ratio of **NIR-1** ($I_{978\ nm}/I_{1056\ nm}$) and the concentration of felodipine. The error bars are the standard deviation of three parallel experiments.

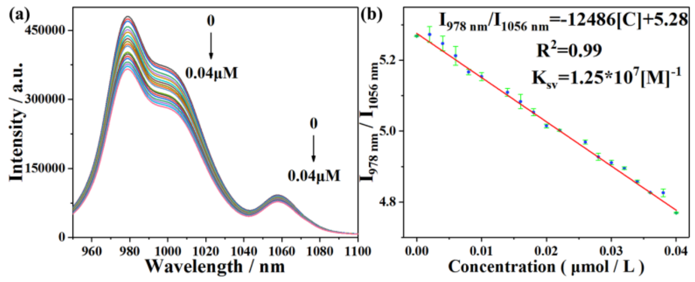

Supplementary Fig. 7 (a) Luminescent spectra of the **NIR-1** (0.2 g/L, 21 cycles) with the addition of felodipine in different concentration (0-40 nM) when excited at 330 nm, the slit is 12 nm; (b) The linear relationship between fluorescent intensity ratio of **NIR-1** ($I_{978\ nm}/I_{1056\ nm}$) and the concentration of felodipine. The error bars are the standard deviation of three parallel experiments.

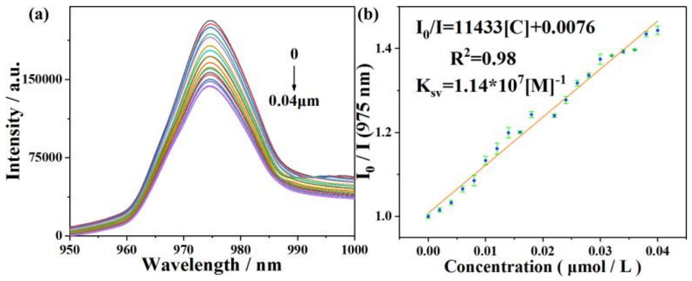

Supplementary Fig. 8 (a) Luminescent spectra of Yb-MOF in DMF solution with the addition of felodipine in different concentration (from 0-40 nM), the exciting position is 330 nm, the slit is 12 nm; (b) The linear relationship between fluorescent intensity ratio of Yb-MOF and the concentration of felodipine. The error bars are the standard deviation of three parallel experiments.

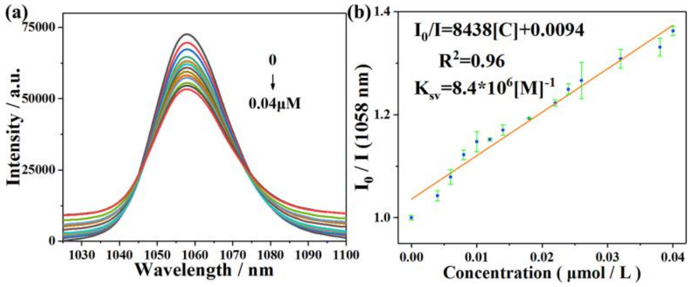

Supplementary Fig. 9 (a) Luminescent spectra of Nd-MOF in DMF solution with the addition of felodipine in different concentration (from 0-40 nM), the exciting position is 330 nm, the slit is 12 nm; (b) The linear relationship between fluorescent intensity ratio of Nd-MOF and the concentration of felodipine. The error bars are the standard deviation of three parallel experiments.

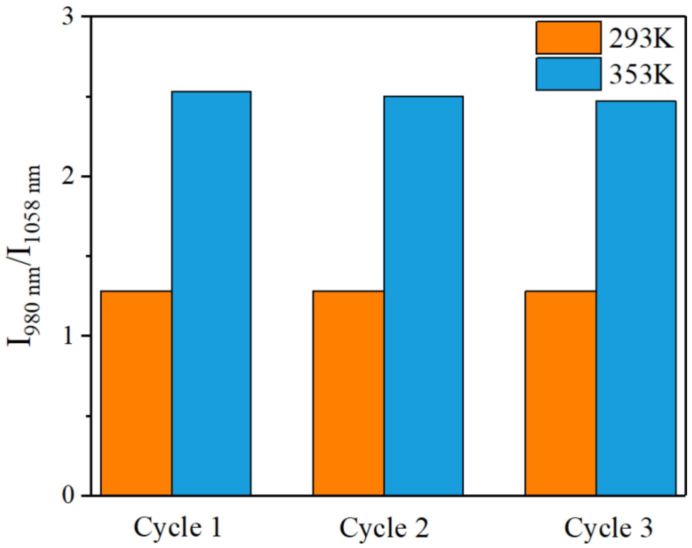

Supplementary Fig. 10 Luminescent intensity ratio of **NIR-1** under different temperature under three parallel experiments

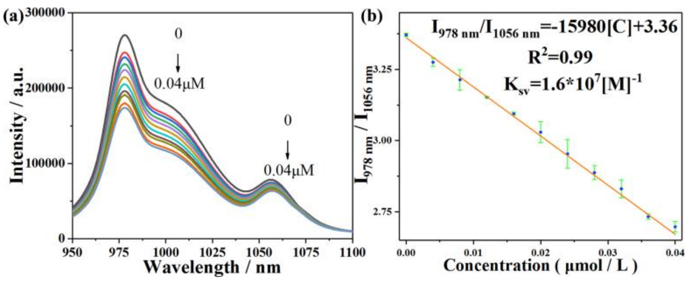

Supplementary Fig. 11. (a) Luminescent spectra of the **NIR-1** (0.2 g/L, 14 cycles) with the addition of the mixture of three drugs (felodipine, bisoprolol, carvedilol) in different concentration (0-40 nM) when excited at 330 nm, the slit is 12 nm; (b) The linear relationship between fluorescent intensity ratio of **NIR-1** ($I_{978\ nm}/I_{1056\ nm}$) and the concentration of the mixture of three drugs. The error bars are the standard deviation of three parallel experiments.

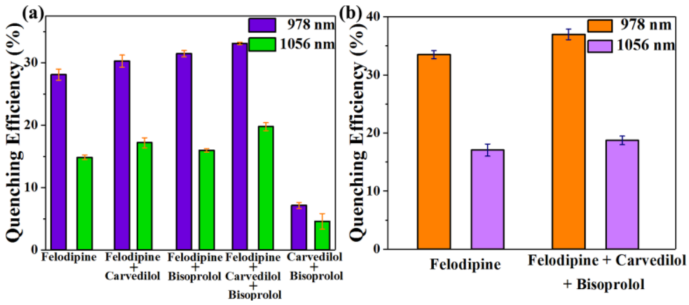

Supplementary Fig. 12. (a) The quenching efficiency (978 nm and 1056 nm) when different drugs solutions (40 nM) were added into **NIR-1** suspensions (0.2 g/L), respectively (exciting position is 330 nm); (b) when different unkown concentration of drugs solutions (50 nM) were added into **NIR-1** suspensions (0.2 g/L), which also enhance the quenching efficiency (978 nm and 1056 nm) (exciting position is 330 nm). The error bars are the standard deviation of three parallel experiments.

Supplementary Table 1 Different element content analyzed in the energy dispersive X-ray microanalysis system (EDS).

Element	Atomic Fraction (%)
C	16.21
N	2.92
O	56.89
Si	5.97
Fe	17.62
Nd	0.08
Yb	0.31
